# Abnormal autophagy, ubiquitination, inflammation and apoptosis are dependent upon lysosomal storage and are useful biomarkers of mucopolysaccharidosis VI

**DOI:** 10.1186/1755-8417-2-4

**Published:** 2009-06-16

**Authors:** Alessandra Tessitore, Marinella Pirozzi, Alberto Auricchio

**Affiliations:** 1 Telethon Institute of Genetics and Medicine (TIGEM), Naples, Italy; 2 Medical Genetics, Department of Pediatrics, 'Federico II' University, Naples, Italy

## Abstract

**Background:**

Lysosomal storage diseases are characterized by intracellular accumulation of metabolites within lysosomes. Recent evidence suggests that lysosomal storage impairs autophagy resulting in accumulation of polyubiquitinated proteins and dysfunctional mitochondria, ultimately leading to apoptosis. We studied the relationship between lysosome storage and impairment of different intracellular pathways and organelle function in mucopolysaccharidosis VI, which is characterized by accumulation of dermatan sulfate and signs of visceral and skeletal but not cerebral involvement.

**Results:**

We show lysosomal storage, impaired autophagy, accumulation of polyubiquitinated proteins, and mitochondrial dysfunction in fibroblasts from mucopolysaccharidosis VI patients. We observe similar anomalies, along with inflammation and cell death, in association with dermatan sulfate storage in the visceral organs of mucopolysaccharidosis VI rats, but not in their central nervous system where dermatan sulfate storage is absent. Importantly, we show that prevention of dermatan sulfate storage in the mucopolysaccharidosis VI rat visceral organs by gene transfer results in correction of abnormal autophagy, inflammation, and apoptosis, suggesting that dermatan sulfate accumulation impairs lysosomal ability to receive and degrade molecules and organelles from the autophagic pathway, thus leading to cell toxicity.

**Conclusion:**

These results indicate that the non-lysosomal degradation pathways we found activated in mucopolysaccharidosis VI can be both targets of new experimental therapies and biomarkers for follow-up of existing treatments.

## Background

Lysosomal storage diseases (LSDs) are severe disorders mostly inherited as autosomal recessive traits in which a lysosomal enzyme defect causes intracellular accumulation of cellular debris within the lysosomes [1]. Little is known about the molecular pathways underlying pathology in LSDs. Degradation and recycling of the building blocks of organelles, proteins, and other cytoplasm components is required for the maintenance of cellular homeostasis [2]. Two general mechanisms are used for large-scale degradation of components of the cytoplasm; short-lived regulatory proteins are degraded via the ubiquitin-proteasome system, and long-lived structures and proteins are targeted to the lysosome by autophagy [2]. Several forms of autophagy have been described [3]. In macroautophagy, henceforth referred to as autophagy, double-membrane vesicles called autophagosomes sequester part of the cytoplasm and then fuse with lysosomes to form hybrid-like organelles called autophagolysosomes [3]. Several proteins are implicated in the formation of autophagosomes. Beclin-1 (BCN1, homologue of yeast ATG6), a protein of the Class III phosphatidylinositol 3 kinase (PI3K) complex, mediates autophagy induction [2]. The microtubule-associated protein 1 light chain 3 (LC3I, homologue of yeast ATG8) is cleaved at its carboxy-terminal, and further modified to the lipid-conjugated LC3II, which is associated to autophagosome membranes [2,4]. In particular, the ratio between the two forms of LC3 (measured as LC3II/LC3I) correlates with the number of autophagosomes [4]. Perturbation of autophagy (that is, blocking of the fusion of autophagosomes to lysosomes, or an increased number of autophagosomes) results in prolonged nutrient starvation, accumulation of toxic intracellular ubiquitin-related protein aggregations which contain polyubiquitinated proteins, and the critical multifunctional protein p62/A170/sequestosome1 (SQSTM1; hereafter referred to as p62) [5,6], and dysfunctional mitochondria, ultimately leading to over-production of reactive oxygen species (ROS), inflammation, and cell death [7]. Abnormal autophagy has been described in human skin fibroblasts and mice models of LSDs, such as Niemann-Pick C1 (NPC1) [8], Danon disease [9], neuronal ceroid lipofuscinosis 2 [10], Pompe disease [11], mucolipidosis type IV [12-14], multiple sulfatase deficiency [15], mucopolysaccharidosis type IIIA [15], and GM1 gangliosidosis [16], indicating that LSDs might be considered as 'disorders of autophagy'. Recently, a model has been proposed suggesting that lysosomal accumulation of undegraded substrates results in defective fusion between autophagosomes and lysosomes [15,17], which, in turn, leads to a progressive accumulation of poly-ubiquitinated protein aggregates and of dysfunctional mitochondria, eventually leading to cell death [17,18]. However, the evidence that substrate accumulation is the primary mediator of these anomalies is still missing.

Mucopolysaccharidosis VI (MPS VI), also known as Maroteaux-Lamy syndrome, is caused by deficiency of the lysosomal enzyme *N-*acetylgalactosamine-4-sulfatase (arylsulfatase B, ARSB) [19]. ARSB hydrolyzes sulfate esters from glycosaminoglycans, mainly dermatan sulfate (DS). ARSB deficiency prevents the sequential degradation of DS leading to its accumulation in various cells and tissues [19]. Clinically, MPS VI is characterized by coarse faces, short stature, dysostosis multiplex, stiffness and functional impairment of joints, hepatosplenomegaly, cardiac valve anomalies and corneal clouding [19]. No clinical signs of central nervous system (CNS) involvement are evident in clinically severe MPS VI [20]. Spontaneous animal models of MPS VI, which closely resemble the human disease, have been described in cats [21], dogs [22], and rats [23]. In agreement with the absence of CNS disease in patients, MPS VI animal models do not show behavioral anomalies nor significant DS accumulation in CNS, although some ultrastructural anomalies in MPS VI cat neurons have been reported [24]. Taking advantage of the difference in storage in visceral organs versus CNS of MPS VI and of the possibility to revert storage by gene transfer, we have studied the relationship between storage and autophagy, polyubiquitination, mitochondrial function, inflammation and apoptosis in MPS VI cells and tissues.

## Results

### Storage accumulation leads to impaired autophagy, abnormal protein ubiquitination and mitochondrial function in human MPS VI cells

**Figure 1:**
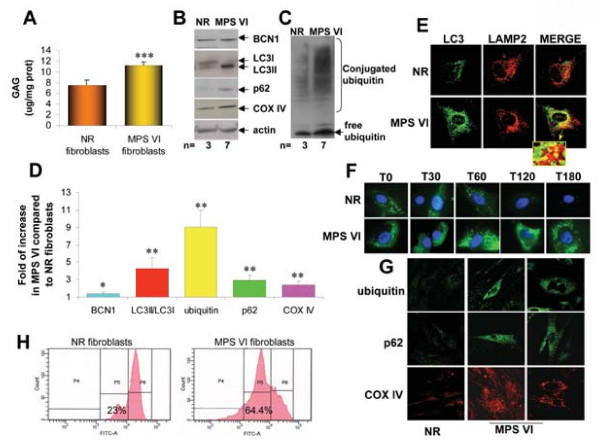
**Impaired autophagy, increased polyubiquitination and dysfunctional mitochondria in human MPS VI fibroblasts**. **(A) **Protein extracts from fibroblasts of three controls (NR fibroblasts) and of seven MPS VI patients (MPS VI fibroblasts) were assessed for glycosaminoglycans (GAGs) accumulation by the quantitative dimethyl-methylene blue method (GAG levels are expressed as μg per mg of proteins: μg GAG/mg prot). **(B and C) **Representative western blot analysis of total lysates from NR and MPS VI fibroblasts blotted with anti-BCN1, -LC3, -p62, -COX IV (B) and -ubiquitin antibodies (C). Normalization of protein loading was performed using anti-actin antibodies (B) and is the same for both panels B and C. **(D) **Quantification of western blot analyses from three independent experiments (mean ± standard error). Values are expressed as fold of increase compared with NR fibroblasts (NR = 1). **(E) **Confocal images of NR and MPS VI fibroblasts stained with anti-LC3 (green) and anti-LAMP2 (red) antibodies. Inserts illustrate the colocalization of the two proteins (merge panel). Magnification: 63×. (**F**) Clearance of loaded Alexa Fluor 488-labeled EGF at various time points (T0, T30, T60, T120 and T180 = 0 min, 30 min, 1 h, 2 h, and 3 h after loading, respectively). Magnification: 100×. **(G) **Human NR and MPS VI fibroblasts were labeled with anti-ubiquitin, -p62 and -COX IV antibodies. Magnification: 63×. **(H) **Measurement of mitochondria membrane potential by flow cytometry analysis of NR and MPS VI fibroblasts after loading with DiOC_6 _and propidium iodide (PI). All experiments were performed in triplicate. *P ≤ 0.05, **P ≤ 0.02, ***P ≤ 0.01.

We hypothesized that excessive DS accumulation alters the lysosomal ability to degrade cytoplasmic components or organelles through autophagy. To test this we initially used primary skin fibroblasts from three controls (normal fibroblasts, NR) and seven MPS VI patients. Measurements of DS accumulation via the quantitative dimethyl-methylene blue method showed significantly higher DS levels in MPS VI cells than in NR cells (Figure 1A). We then sought to determine whether abnormal autophagy occurs in MPS VI cells by analyzing LC3 levels. Western blot analyses of protein lysates from skin fibroblasts showed increased levels of LC3II in MPS VI compared with NR fibroblasts (Figure 1B and 1D), indicating accumulation of autophagosome proteins. Furthermore, confocal microscopy analysis confirmed increased numbers of LC3-positive vesicles in MPS VI compared with NR fibroblasts (Figure 1E) some of which colocalized with the lysosomal marker, lysosome-associated membrane protein-2 (LAMP2), as indicated by the presence of yellow signal in the merged panel (Figure 1E, see also insert). Our data show that the extent of LAMP2/LC3 colocalization is similar between MPS VI and NR fibroblasts, thus suggesting that autophagosome-lysosome fusion is not completely blocked in MPS VI fibroblasts (Figure 1E). To understand whether the increase in autophagic markers observed in MPS VI cells is due to the deficient ability of lysosomes to recycle metabolites, we compared the Epidermal Growth Factor (EGF)/EGF Receptor (EGFR) turnover in MPS VI and NR fibroblasts based on the knowledge that binding of EGF to EGFR induces ubiquitination, rapid internalization and degradation of both ligand and receptor via the lysosomal pathway [25,26]. Loading of EGF on NR fibroblasts resulted in its almost complete clearance in less than two hours, while EGF signal was still persistent in MPS VI fibroblasts three hours after loading (Figure 1F). These results were additionally confirmed by time lapse analysis of NR and MPS VI fibroblasts loaded with both EGF and lyso-tracker; EGF delivery to lysosomes and subsequent degradation was slower in MPS VI compared with NR fibroblasts (Additional files 1 and 2). These results suggest that lysosomal ability to recycle metabolites is impaired in MPS VI presumably resulting in accumulation of autophagosomes. We then postulated that impaired autophagy observed in MPS VI fibroblasts could in turn result in accumulation of ubiquinated proteins similarly to what has been observed in other LSDs [10,13-15]. Western blot analysis with anti-ubiquitin antibodies of fibroblast lysates showed increased ubiquitin levels in MPS VI cells when compared with NR cells (Figure 1C and 1D). Ubiquitin was accumulated in ubiquitin-positive inclusions as assessed by immuno-fluorescence analysis (Figure 1G). The accumulation of ubiquitinated proteins in MPS VI cells occurred in the presence of normal proteasome function as demonstrated by the *in vitro *analysis of proteasome activity (data not shown). This suggests that the increased ubiquitin levels detected are secondary to the defective autophagy observed rather than to a primary proteasome impairment. In agreement with this finding, we found significant p62 accumulation in MPS VI fibroblasts compared with NR by both western blot and immuno-fluorescence analyses (Figure 1B, D, and 1G). To test whether impaired lysosomal function in MPS VI fibroblasts affects autophagy of mitochondria, resulting in accumulation of dysfunctional mitochondria, we measured the levels of the mitochondrial marker COX IV by western blot and by immuno-fluorescence analyses in MPS VI fibroblasts and found it increased compared with controls (Figure 1B, D, and 1G). In addition, using the mitochondria-specific voltage dependent dye DiOC_6 _[27] we detected a reduction in the mitochondrial membrane potential in MPS VI compared with NR fibroblasts in both normal (data not shown) and starved conditions (Figure 1H), measured as increase in DiOC_6 _fluorescence (from 23 ± 2.5% in NR cells to 56.9 ± 4.2% in MPS VI cells, *P *= 0.05). These results imply volume changes in MPS VI mitochondria, indicating that they are dysfunctional. Finally, the modest increase in BCN1 levels (Figure 1B and 1D) observed in MPS VI fibroblasts suggests a positive feedback on autophagy triggered by the inability of lysosomes to receive and degrade macromolecules from the autophagic membrane-trafficking pathway.

### Dermatan sulfate accumulation in visceral organs of MPS VI rats results in abnormal autophagy, ubiquitination, mitochondrial function, inflammation, and apoptosis

**Figure 2:**
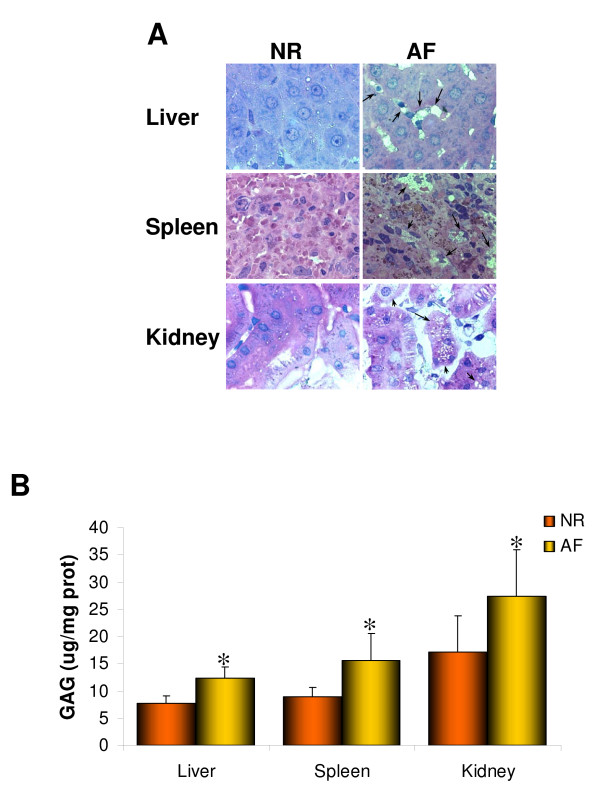
**Glycosaminoglycan (GAG) accumulation in visceral organs of MPS VI rats**. **(A) **Toluidine blue staining of semi-thin sections (1 μm thick) shows vacuoles filled with storage (arrows) in lysosomes of cells from liver, spleen, and kidney of 6-month-old MPS VI affected (AF) rats, which were absent in the same tissues of normal (NR) rats. Magnification: 100×. **(B) **Quantitative measurement of GAG accumulation via dimethyl-methylene blue method on tissue lysates from liver, spleen, and kidney of AF and NR rats. (GAG levels are expressed as μg per mg of proteins: μg GAG/mg prot). *P ≤ 0.01.

**Figure 3:**
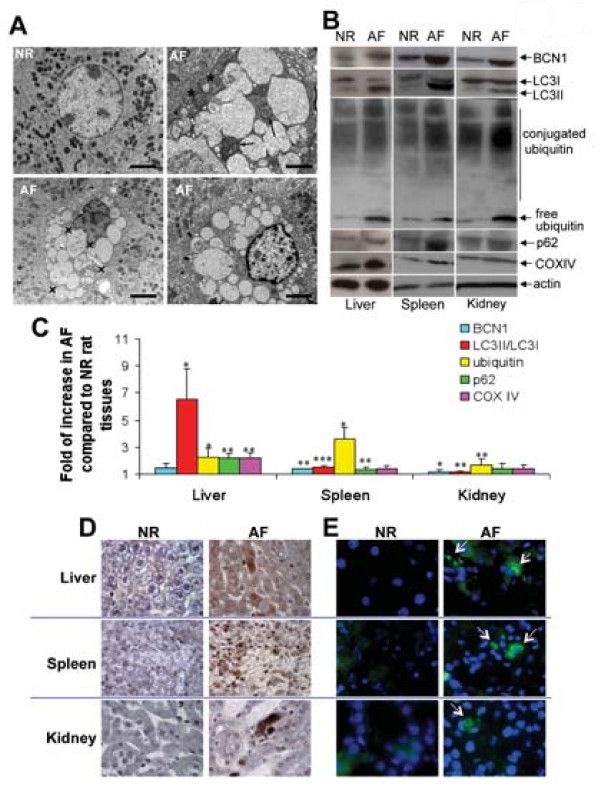
**Impaired autophagy, increased polyubiquitination and dysfunctional mitochondria in visceral organs of MPS VI rats**. **(A) **Electron-microscopic analysis of liver cells from 6-month-old normal (NR) or affected (AF) rats. Arrows, arrowheads and 'x' symbols point at autophagic vescicles (AVs) with different morphology (see *Results *for description). Asterisks indicate accumulated mitochondria with altered morphology, which are sometimes included in AVs. Scale bars = 2.2 μm. **(B) **Representative western blot analysis of tissue lysates from NR and AF rat liver, spleen, and kidney blotted with anti -BCN1, -LC3, -ubiquitin, -p62, -COX IV and -actin antibodies. **(C) **Quantification of western blot analyses. Values are expressed as fold of increase compared with NR rat tissues (NR = 1), and are the mean ± standard error of three independent experiments. **(D) **Immuno-histochemical analysis with anti-ubiquitin antibodies and **(E) **immuno-fluorescence analyses with anti-p62 antibodies of liver, spleen, and kidney sections from 6-month-old NR and AF rats. Magnification 100×. *P ≤ 0.05, **P ≤ 0.01, ***P ≤ 0.005.

To confirm the results observed in MPS VI human fibroblasts *in vivo *we studied a rat model of MPS VI which shows severe signs of visceral and skeletal but not of CNS involvement. We observed that *in vivo *DS accumulates in peripheral tissues. Storage was detected in liver, spleen, and kidney using toluidine blue staining of semi-thin sections (Figure 2A, arrows) and using the quantitative dimethyl-methylene blue assay (Figure 2B). We then tested whether DS accumulation in lysosomes correlates with abnormal autophagy in MPS VI rat tissues. Electron-microscopy analysis of liver sections from 6-month-old normal (NR) and MPS VI affected (AF) rats revealed a higher number of autophagic vacuoles (AVs) in AF rat sections compared with NR (Figure 3A). The autophagic vacuoles appear as double-layered vacuoles encircled by ER-like membrane saccules, and contain cytoplasmic organelles together with part of the cytoplasm. AV morphology showed abnormal autophagic figures, with various morphologic features reflecting different stages of the disease [28,29]. Some AVs (arrowheads) showed normal appearance similar to that observed following autophagy induction when no impairment of autophagosome-lysosome fusion occurs [28,29]. Other AVs (x symbols) have organellar structures accumulated within swollen vescicular compartments, which is typical of AVs formed after a short exposure to drugs which block autophagy [28,29]. This observation may reflect a later stage of the disease when impairment in autophagosome-lysosome fusion occurs because of the inability of the engulfed lysosomes to degrade their content. Finally, if metabolites persist in AVs for long enough, their content becomes electron-dense and compact (black arrows) [28,29]. Similarly to that observed in MPS VI human fibroblasts, western blot analyses of liver, spleen, and kidney lysates demonstrate increased levels of LC3II (Figure 3B and 3C) in AF compared with NR rat tissues, possibly indicating that lysosomal accumulation of DS results in impairment of the autophagic pathway and in accumulation of AVs *in vivo*. In addition, increased levels of ubiquitin and of p62, as detected by western blot (Figure 3B and 3C) and by immune-histochemistry (Figure 3D) or immuno-fluorescence (Figure 3E) analyses, suggest the formation of intracellular ubiquitin-aggregates as consequence of impaired autophagy. Accordingly, increased levels of COX IV, measured by western blot analysis, suggest the accumulation in visceral AF organs of mitochondria (Figure 3B and 3C), some of which appeared damaged, as evidenced by the abnormal deposition of electron-dense multilayered material (Figure 3A, both arrowheads, for mitochondria included in AVs, and asterisks). Similarly to that observed in MPS VI fibroblasts, a slight elevation in BCN1 levels (Figure 3B and 3C) suggests a positive feedback on autophagy induced by lysosome overloading.

**Figure 4:**
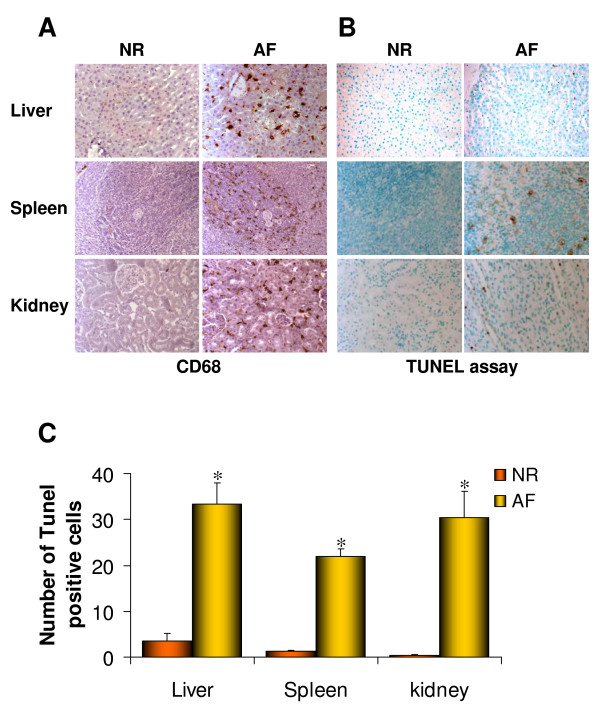
**Inflammation and apoptosis in visceral organs from MPS VI affected rats**. **(A) **Immuno-histochemical analysis with anti-CD68 antibodies and **(B) ***in situ *TUNEL analyses of liver, spleen, and kidney sections from 6-month-old normal (NR) and affected (AF) rats. Magnification: 40×. **(C) **Apoptotic cell count performed on liver, spleen, and kidney sections from 6-month-old NR and AF rats. *P ≤ 0.0001.

We then asked whether engulfment of cells, due to DS accumulation and impaired AV recycling, results in activation of inflammation and eventually in cell death. Abundant CD68-positive monocyte/macrophage cells were detected via immuno-histochemical analysis of 6-month-old liver, spleen, and kidney sections of AF compared with NR rats (Figure 4A and [30]). We also performed TUNEL assay in the same sections detecting apoptotic cells in AF rats, which were almost completely absent in NR rats (Figure 4B and 4C and [30]).

**Figure 5:**
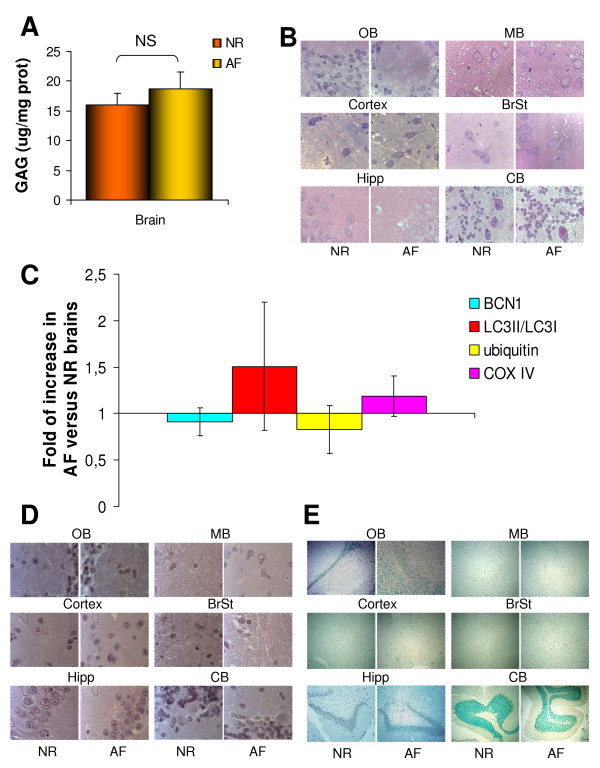
**Normal autophagy, ubiquitination, mitochondrial function, absence of inflammation, and apoptosis in MPS VI CNS without significant DS storage**. **(A) **Quantitative measurement of glycosaminoglycan (GAG) accumulation via dimethyl-methylene blue method on brain lysates of 6-month-old affected (AF) and normal (NR) rats. (GAG levels are expressed in μg per mg of proteins: μg GAG/mg prot). **(B) **Toluidine blue staining of semi-thin sections (1 μm thick) of brain from 6-month-old AF and NR rats. Magnification: 100×. **(C) **Quantification of western blots performed on brain lysates from 6-month-old NR and AF rats. Values are expressed as fold of increase compared with NR rat brains (NR = 1) and are the mean ± standard error of three independent experiments. Western blot with anti-p62 antibodies did not allow the detection of any band in the brain of either NR or AF rats. **(D) **Immuno-histochemical analysis with anti-CD68 antibodies and **(E)***in situ *TUNEL analysis of brain sections from 6-month-old NR and AF rats. Magnification: 100× and 20×, respectively. NS = not statistically significant; OB = olfactory bulb; MB = middle brain; BrSt = brain stem; Hipp = hippocampus; CB = cerebellum.

**Figure 6:**
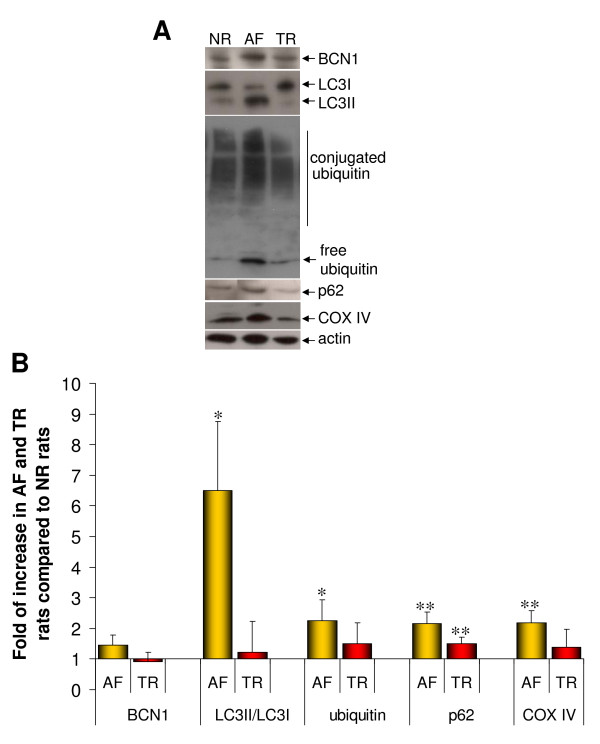
**Reduction of autophagic, ubiquitination, and mitochondrial anomalies in livers of MPS VI rats following arylsulfatase B gene transfer and DS normalization**. **(A) **Representative western blot analysis of tissue lysates from livers of 6-month-old NR and MPS VI rats treated (TR) or not (AF) with AAV. Membranes were blotted with anti-BCN1, -LC3, -ubiquitin, -p62, -COX IV and -actin antibodies. **(B) **Quantification of western blots. Values are expressed as fold of increase compared with NR rat tissues (NR = 1) and are the mean of three independent experiments. *P ≤ 0.05, **P ≤ 0.01.

MPS VI rats, similarly to patients, do not show signs of CNS involvement, strongly suggesting that substrate accumulation does not occur in this organ. This provides the unique opportunity to determine whether the abnormal pathways observed in MPS VI visceral organs only occur in the presence of DS accumulation and therefore to assess whether the phenotype observed in cells and affected organs is due to lysosomal storage. We initially measured DS accumulation and the presence of vacuolated cells in CNS of 6-month-old MPS VI rats. Quantitative measurement using the dimethyl-methylene blue method showed comparable levels of DS in CNS of AF and NR rats (Figure 5A). Similarly, electron microscopy (data not shown) and toluidine blue staining of semi-thin brain sections from either AF or NR rats (Figure 5B) did not show the presence of cellular vacuolization. Consistent with absence of CNS lysosomal storage, western blot analysis of brain lysates revealed normal BCN1, LC3II, ubiquitin, and COX IV levels, indicating normal autophagy, ubiquitination, and mitochondrial function in neuronal MPS VI cells (Figure 5C). Immuno-histochemical analysis using anti-ubiquitin antibodies as well as immuno-fluorescence analysis using anti-p62 antibodies showed normal patterns of expression in CNS of AF rats (data not shown). Similarly, no CD68 and TUNEL positive cells were detected in CNS of either AF or NR rats (Figure 5D and 5E, respectively). These data clearly indicate that the presence of abnormal degradation pathways, inflammation, and apoptosis is strongly associated with lysosomal storage in MPS VI tissues. To additionally prove that DS storage is upstream of the abnormalities observed in visceral organs *in vivo*, we tested, using somatic gene transfer, whether DS clearance rescues the phenotype observed thus resulting in the normalization of autophagy, ubiquitination, mitochondrial function, and ultimately inflammation and apoptosis in the affected tissues of AF rats. We recently reported that systemic administration of adeno-associated viral (AAV) vectors expressing ARSB in newborn MPS VI rats results in therapeutic levels of circulating ARSB and in a significant decrease of DS storage in visceral organs [30]. Using the same protocol described in [30], MPS VI rats were injected at birth (post-natal day 3 to 5) with 4.1 × 10^13 ^genome copies/kg of AAV2/8 TBG-ARSB in the temporal vein (indicated as TR). Six months after injection rats were sacrificed and tissues collected for analysis. Controls included age-matched NR and non-treated AF rats. In order to assess impaired autophagy, ubiquitination, and mitochondrial dysfunction we analyzed the levels of marker proteins (BCN1, LC3II, ubiquitin, p62, and COX IV). Western blot analyses of liver (Figure 6A and 6B), spleen, and kidney lysates (data not shown) from NR and TR animals showed normal levels of all markers tested as opposed to AF lysates. These results indicate that storage reduction normalizes lysosomal-associated alterations, indicating that the molecules studied can be used as biomarkers to assess the efficacy of preventive and therapeutic interventions.

## Discussion

Despite the differences in the type and the amount of metabolites accumulated in LSDs as well as the cells or tissues where storage occurs, the clinical and pathological manifestations are to some extent similar among LSDs, thus suggesting common mechanisms of disease triggered by different genetic defects [8-16,30-36]. Identification of critical cellular mediators within these processes may help develop therapies to target them and biomarkers for follow-up of disease progression and therapeutic intervention.

A growing body of evidence suggests that lysosomal storage leads to reduced functionality of lysosomes and consequent autophagy deregulation [15-17]. In this study, we showed impaired autophagy with increased levels of autophagic proteins, increased polyubiquitination and abnormal mitochondrial function in human MPS VI fibroblasts as well as in affected tissues of an MPS VI rodent model. *In vivo*, this was associated with inflammation and apoptosis. This adds to what has been observed in other LSDs [8-16], where abnormal autophagy has been described, proving that common mechanisms are downstream of different genetic defects in LSDs. The increased amount of autophagosomes in MPS VI fibroblasts might be explained by the inability of lysosomes engulfed with DS to recycle. Indeed, disruption of lysosome function inhibits the fulfillment of autophagy with consequent massive accumulation of autophagosomes. This is indirectly suggested by the slow EGF/EGFR turnover observed in MPS VI fibroblasts. Interestingly, the EGF/EGFR complex is recycled in lysosomes by cathepsin B [26]. Glycosaminoglycans are reported to inhibit cathepsin activity [37] and cathepsin-activity deficiency results in impaired autophagy [38].

Alterations in the autophagy-lysosomal degradation pathway have been linked to normal brain aging [39], to age-related neurodegenerative diseases including Alzheimer's (AD) [40], Parkinson's (PD) [41], and Huntington's (HD) diseases [42] in addition to several LSDs [8-16]. Since deregulation of autophagy is associated with disease progression, it has been speculated that modulating autophagy activity may result in therapeutic efficacy. Enhancement of autophagy (that is, through treatment with rapamycin) may help clear aggregated proteins, as observed in neurodegenerative disorders [43]; however, because autophagy relies on intact lysosomes for appropriate autophagosome-lysosome fusion, the progressive impairment of lysosome function, as it occurs in LSDs, may reverse any long-term benefits derived from the over-stimulation of autophagy, resulting in nutrient starvation and ultimately in autophagic cell death [44]. Indeed, although induction of autophagy in AD has an initial protective role, long-term over-stimulation of autophagy induces neuronal cell death. Conversely, inhibiting autophagy either pharmacologically or via RNA interference of specific genes significantly attenuates cell death in AD and PD, respectively [40,45]. Therefore, agents that attenuate autophagy might be similarly useful for treatment of LSDs with increased levels of autophagic markers, that is, NPC, GM1, and now, based on the results of this study, MPS VI.

Although additional studies are required to prove the mechanisms linking autophagy impairment to polyubiquitination anomalies, mitochondrial dysfunction, inflammation, and apoptosis in MPS VI, some hypotheses can be drawn. For instance, mitochondria produce metabolic energy and free radicals (that is, reactive oxygen species (ROS)), serve as biosensors for oxidative stress, and eventually become effectors of apoptosis [12,46]. In turn the accumulation of fragmented mitochondria we have observed in MPS VI cells and tissues may cause increasing oxidative stress resulting in inflammation, which finally triggers cell death responses as observed in different disorders [47]. Most importantly, our data support a strong association between lysosomal storage and abnormal degradation pathways, inflammation, and apoptosis *in vivo*. These were present in liver, spleen, and kidney of MPS VI rats where we detect significant DS storage and were absent in the CNS of the same animals where DS storage is absent. In addition, when DS storage is reduced in liver, spleen, and kidney following somatic AAV-mediated gene transfer, levels of autophagic markers, polyubiquitinated proteins, fragmented mitochondria, inflammation, and apoptosis are normalized, demonstrating a therapeutic efficacy on autophagy deregulation and mitochondrial dysfunction in addition to apoptosis and inflammation, as previously described [30]. Similar data have been reported in cartilage and synovial tissues of MPS VI rats, where authors ascribe the onset of inflammation and apoptosis to glycosaminoglycan storage [48,49]. Moreover, autophagic markers, polyubiquitinated proteins, fragmented mitochondria, inflammation, and apoptosis can be used as biomarkers for follow-up of disease progression. This may be relevant to understanding the clinical history of the disease and to defining the endpoint assessment of therapeutic regimens such as enzyme replacement therapy, bone marrow transplantation, and gene therapy.

## Conclusion

In this paper we have studied the relationship between storage and secondary events, such as autophagy, polyubiquitination, mitochondrial function, inflammation, and apoptosis, in MPS VI cells and tissues. We have demonstrated a direct link between substrate storage and abnormal cellular pathways which contribute to the pathophysiology of MPS VI, and we have identified new useful biomarkers for follow-up of disease progression. Our data may help in the development of new therapies which act downstream of the genetic defect in this and other LSDs.

## Methods

### Tissue cultures, animal colonies, and tissue collection

Fibroblasts from MPS VI patients and from normal subjects were grown at 37°C with 5% CO_2_, in RPMI (Gibco-Invitrogen, Grand Island, NY, USA) and 10% fetal bovine serum (FBS, Sigma-Aldrich, St Louis, MO, USA), supplemented with 100 U/ml penicillin, 100 μg/ml streptomycin (Gibco-Invitrogen, USA). The cell lines were used between passage 2 and 8, and maintained at the same passage number in each experiment performed.

MPS VI rats were maintained at the Cardarelli Hospital's Animal House (Naples, Italy) in an appropriate environment according to the Italian Ministry of Health regulation. Normal and affected offspring were obtained and genotyped as previously described [30]. Tissues were collected from 6-month-old rats in accordance to the Italian Ministry of Health guidelines as previously described [30]. Each tissue collected was divided in pieces and fixed for plastic and paraffin embedding or frozen in dry ice for ARSB activity, GAG quantitative assays, and protein extraction.

### Antibodies

Primary antibodies were: rabbit polyclonal anti-LC3 (Novus Biological, Littleton, Colorado, USA), rabbit polyclonal anti-beclin 1 (Santa Cruz Biotechnology, Santa Cruz, California, USA), goat monoclonal anti-LAMP2 (Santa Cruz Biotechnology, USA), mouse monoclonal anti-ubiquitin (Cell Signaling, Danvers, Massachusetts, USA), mouse monoclonal anti-P62/SQSTM1 (BD, Franklin Lakes, New Jersey, USA), mouse monoclonal anti-actin (Sigma-Aldrich, St Louis, Missouri, USA) and rabbit polyclonal anti-COXIV (Cell Signaling, USA). Secondary antibodies were: goat anti-rabbit or anti-mouse conjugated to Alexa Fluor 488 or 594 (Molecular Probes – Invitrogen, Eugene, Oregon, USA). HRP-conjugated anti-mouse or anti-rabbit IgG (Amersham, Freiburg, Germany); biotinylated donkey anti-rabbit (Jackson ImmunoReasearch, West Grove, Pennsylvania, USA).

### Protein extraction and western blot analysis

Cells were lysed in cold lysis buffer (50 mM Tris-HCl, pH 7.5; 150 mM NaCl; 0.5% DOC; 0.5% NP-40; 2% sodium azide) in the presence of protease (Roche Diagnostics, Mannheim, Germany) and phosphatase (cocktails I and II by Sigma-Aldrich, St Louis, Missouri, USA) inhibitors for 30 min on ice. Tissue samples (50 μg) were homogenized in 3 volumes of lysis buffer and proteins were quantified using the BCA protein assay reagent kit (Pierce Chemical Co, Rockford, Illinois, USA) according to the manufacturer's instructions. Primary and (HRP)-conjugated antibodies were diluted in 5% milk. Bands were visualized using the ECL detection reagent (Pierce Chemical Co, USA).

### Confocal microscopy

A Leica inverted DMIRE2 epifluorescence microscope equipped with a Leica laser-scanning confocal image system TCS SP2 AOBS (Leica Microsystems, Heidelberg, Germany) was used for data acquisition. Samples were excited with a 488 nm Ar laser and 594 nm He-Ne laser. Samples were vertically scanned from the bottom coverslip with a total depth of 50 mm and a 63× (1.32 NA) HP PLAPO oil-immersion objective. A total of 10 z-line scans with a step distance of 0.2 mm was collected and maximum intensity projections were generated with Leica Confocal Software (Leica Microsystems, Wetzlar, Germany).

### EGF loading, time-lapse microscopy and immuno-fluorescent analysis

For time-lapse microscopy, skin fibroblasts from normal and MPS VI patients were plated in 35-mm glass-bottom dishes (Willco BV, Amsterdam, the Netherlands) and were incubated at 37°C in 5% CO2 for 16 h, after which they where starved for 2 h with no-serum medium. Following starvation, cells were loaded with 1 μg of Alexa Fluor 488-labeled EGF (Molecular Probes, Invitrogen, USA) and 0.1 μM LysoTacker Red DND-99 (Molecular Probes, Invitrogen, USA) for 1 h at 4°C. After incubation, cells were washed three times with 1 × PBS and medium was replaced with fresh 10% FBS medium. Cells were mounted on Leica AF6000 LX multiposition advanced fluorescence imaging and live cell analysis system (Leica Microsystems, Wetzlar, Germany). The live imaging was performed using an inverted microscope system (Leica DMI6000; Leica, Heidelberg, Germany) equipped with environment control boxes and digital camera (CCD). Images were acquired in fluorescence (GFP and RFP) and transmission (DIC) channels with a 63× glycerin-immersion objective. Usually, stacks about 10 μm thick, composed of sections separated by 0.22 μm, were taken every 15 min during an average period of 24 h. To avoid fading of the fluorescence, the intensity levels were fixed at less than position 2. The 4D captured images thus obtained were deconvoluted using the blind algorithm and adjusted using the brightness switch implemented in the software package AF6000 (Leica, Heidelberg, Germany). Maximum intensity projection of Z-stacks was done for 4D images. Online material (Additional files 1 and 2) contains live-cell imaging.

For immuno-fluorescent microscopy, skin fibroblasts from normal and MPS VI patients were plated in chamber slides (LabTek International, Naperville, Illinois, USA) and loaded with 1 μg of Alexa Fluor 488-labeled EGF (Molecular Probes, Invitrogen, USA) as described above. After washing, 10% FBS fresh medium was added onto the cells, which were incubated at 37°C in 5% CO2, until fixed at different time points with 4% PFA and mounted with Vectashield with DAPI (Vector Laboratories, Burlingame, California, USA).

### Mitochondrial membrane potential measurements

PBS-washed 1 × 10^6 ^cells were incubated in 1.3 nM DiOC6 (Sigma-Aldrich, St Louis, Missouri, USA) and 1 mg/ml propidium iodide (PI, Sigma-Aldrich, St Louis, Missouri, USA) for 15 min at 37°C. After washing, cells were suspended in 1 ml PBS (pH 7.4) and were subsequently analyzed using flow cytometry. PI was used as counterstain to exclude dead cells from the analyses. At least 10,000 cells in both normal and MPS VI were analyzed for each sample. The experiments were performed in triplicate, and all statistical analyses were performed using Stat-View 5.0 (Statsoft, Tulsa, Oklahoma, USA).

### Assay of proteasome activity

20S proteasome activity was assayed on total lysates of cultured fibroblasts and rat tissues (liver, spleen, kidney, and brain) using the Chemicon (Temecula, California, USA) assay kit, according to the manufacturer's recommended protocol.

### Semi-thin sections and immuno-histochemistry

For semi-thin sections, tissues were collected and fixed in 2.5% PFA and 2% glutaraldehyde for 12 h; post-fixed in osmium tetroxide, block stained with 1% uranyl acetate, dehydrated in ethanol, and embedded in plastic. Semi-thin sections (1 μm thick) were stained with 0.1% toluidine blue (Fisher Scientific, Pittsburgh, Pennslyvania, USA). For immuno-histochemistry, tissues were fixed in 4% PFA for 12 h and embedded in paraffin (Sigma-Aldrich, St Louis, Missouri, USA) after their dehydration with a 70% to 100% ethanol gradient. Finally, the tissues were sectioned to 5 μm serial sections on a microtome. CD68 staining was performed as previously described [30].

### Electron microscopy analysis

Animal tissues (brains and livers) were fixed with 1% glutaraldehyde, washed, stained with uranylacetate and OsO4, dehydrated in ethanol and embedded in Epon. Resin blocks were sectioned using Ultracut UCT ultramicrotome (Leica Microsystems, Wetzlar, Germany). EM images were acquired from thin sections under a Philips Tecnai-12 electron microscope (Philips, Eindhoven, the Netherlands) using an ULTRA VIEW CCD digital camera (Soft Imaging Systems GmbH, Münster, Germany).

### Quantitative analysis of GAG accumulation in tissues and urine

The urine and the protein extracts were assayed with the dimethylmethylene blue-based spectrophotometry of glycosaminoglycans. Briefly, tissues were homogenized in water and centrifuged. After protein quantification, 10 μg of protein extracts or 5 μl of urine were used for the colorimetric assay as previously described [30]. The samples were read at 520 nm and the GAG concentrations were determined using the dermatan sulfate standard curve (Sigma-Aldrich, St Louis, Missouri, USA). Tissue GAG was expressed as μg GAG/mg protein.

### TUNEL assay

TUNEL assay was performed on 5-μm fixed liver sections. Apoptotic cells were detected by using the ApopTag In Situ Apoptosis Detection Kit (Chemicon-Millipore, Temecula, California, USA), as previously described [30].

## Statistical analysis

Data were analyzed by one-way ANOVA (analysis of variance) and are expressed as the mean ± standard error. For all statistical testing, a *P *value less than 0.05 was considered significant.

## Abbreviations

AAV: adeno-associated viral; AD: Alzheimer's disease; ANOVA: analysis of variance; ARSB: arylsulfatase B; AV: autophagic vacuole; CNS: central nervous system; DS: dermatan sulfate; EGF: Epidermal Growth Factor; EGFR: Epidermal Growth Factor Receptor; HD: Huntingdon's disease; LAMP2: lysosome-associated membrane protein-2; LSD: lysosomal storage disease; MPS VI: mucopolysaccharidosis VI; PD: Parkinson's disease; ROS: reactive oxygen species.

## Competing interests

The authors declare that they have no competing interests.

## Authors' contributions

AT performed all the experiments and contributed to the design of the study, the interpretation of results, and the draft of the manuscript. MP helped in the acquisition of confocal microscopy images and the development of the time-lapse microscopy data. AA conceived the study and participated in its design, the interpretation of results, and the drafting of the manuscript. All authors have read and approved the final manuscript.

## Supplementary Material

Additional file 1 **Epidermal Growth Factor (EGF)/EGF Receptor (EGFR) turnover in NR fibroblasts**. Normal skin fibroblasts were loaded with LysoTracker (red) and EGF (green) to follow the EGF/EGFR turnover via lysosomal degradation. Image sequences were recorded at one frame per 15 min, played at 3 frames/sec, and cover 20 sec (5 h). Time is indicated in the upper left corner and is hours:minutes:seconds. Scale bar = 20 μm. Click here for file

Additional file 2 **Epidermal Growth Factor (EGF)/EGF Receptor (EGFR) turnover in MPS VI fibroblasts**. MPS VI skin fibroblasts were loaded with LysoTracker (red) and EGF (green) to follow the EGF/EGFR turnover via lysosomal degradation. Image sequences were recorded at one frame per 15 min, played at 3 frames/sec, and cover 20 sec (9 h). Time is indicated in the upper left corner and is hours:minutes:seconds. Scale bar = 20 μm. Click here for file
